# The effectiveness of nano chemotherapeutic particles combined with mifepristone depends on the PR isoform ratio in preclinical models of breast cancer

**DOI:** 10.18632/oncotarget.1922

**Published:** 2014-04-27

**Authors:** Gonzalo Sequeira, Silvia I Vanzulli, Paola Rojas, Caroline Lamb, Lucas Colombo, María May, Alfredo Molinolo, Claudia Lanari

**Affiliations:** ^1^ Institute of Experimental Biology and Medicine, IBYME-CONICET, Buenos Aires, Argentina; ^2^ National Academy of Medicine, Buenos Aires, Argentina; ^3^ Instituto Roffo, Buenos Aires, Argentina; ^4^ Oral and Pharyngeal Cancer Branch, NIDCR, NIH, Bethesda, USA

**Keywords:** breast cancer, PR isoforms, doxorubicin, paclitaxel, mifepristone, mammary carcinomas, Nab-paclitaxel, pegylated doxorubicin liposomes

## Abstract

There is clinical and experimental evidence suggesting that antiprogestins might be used for the treatment of selected breast cancer patients. Our aim was to evaluate the effect of albumin-bound paclitaxel (Nab-paclitaxel) and pegylated doxorubicin liposomes (PEG-LD) in combination with mifepristone (MFP) in experimental breast cancer models expressing different ratios of progesterone receptor (PR) isoforms A and B. We used two antiprogestin-responsive (PRA>PRB) and two resistant (PRA<PRB) murine mammary carcinomas growing in BALB/c, GFP-BALB/c or nude mice, along with human T47D-YA and T47D-YB xenografts growing in immunocompromised NSG mice. MFP improved the therapeutic effects of suboptimal doses of Nab-paclitaxel or PEG-LD in murine and human carcinomas with higher levels of PRA than PRB. MFP induced tissue remodeling in PRA-overexpressing tumors, increasing the stromal/tumor cell ratio and the number of functional vessels. Accordingly, an increase in nanoparticles and drug accumulation was observed in stromal and tumor cells in MFP-treated tumors. We conclude that MFP induces an increase in vessels during tissue remodeling, favoring the selective accumulation of nanoparticles inside the tumors. We propose that antiprogestins have the potential to enhance the efficacy of chemotherapy in breast tumors with a high PRA/PRB ratio.

## INTRODUCTION

Despite efforts to develop novel therapeutic anticancer agents to block specific signaling pathways, chemotherapy is still the gold standard for advanced breast cancer treatment. The challenge is to increase chemotherapeutic concentrations within the tumor environment while decreasing the general toxicity. Two drugs have been widely used: paclitaxel [1] and doxorubicin [2].

Paclitaxel is a diterpenoid product extracted from *Taxus brevifolia* [3] that causes G2/M cell cycle arrest by promoting microtubule assembly from tubulin and preventing microtubule depolymerization [4;5], inducing apoptosis through phosphorylation and downregulation of the anti-apoptotic proteins BCL-2 and BCL-XL respectively [6-9]. Because paclitaxel has very little aqueous solubility, a Cremophor /ethanol vehicle is used, but its toxicity limits the amount of paclitaxel that can be clinically administered [10-12]. To overcome this problem, an albumin-bound formulation of paclitaxel (Nab-paclitaxel) devoid of any solvents has been developed, and a better response was obtained with the latter when equitoxic doses of free or Nab-paclitaxel were given [13;14].

Doxorubicin is a 14-hydroxylated version of daunorubicin, which is a natural product produced by wild type strains of *Streptomyces* [15]. The clinical usefulness of doxorubicin is limited due to dose-related progressive myocardial damage [16]. To overcome this drawback, different methods of drug delivery were developed, including pegylated liposomes. Due to the leaky vasculature of tumor vessels, pegylated liposomes preferentially distribute to tumors over normal tissue, thus decreasing the incidence rate of cardiotoxicity [17].

Two-thirds of breast cancers express hormone receptors and are subjected to endocrine therapy generally aimed at blocking estrogen receptors (ER) [18]. There is, however, compelling evidence suggesting that progesterone receptors (PRs) may also be used as therapeutic targets [reviewed in [19-20]. Most PR+ tumors express levels of PR isoform A (PRA) higher than those of isoform B (PRB) [21-25] and the presence of PRA may reduce the efficiency of taxanes on mammary tumor growth [26]. We have shown that murine mammary carcinomas with a high PRA/PRB ratio respond to antiprogestin treatment [27-29] and that this is characterized by an increase in stroma [30] and in metalloprotease activity [31].

We hypothesized that in PRA positive tumors, co-treatment of antiprogestins may allow for the use of lower doses of chemotherapeutic agents thereby reducing their side effects. In this study, we show that mifepristone (MFP) treatment improves the therapeutic effect of pegylated doxorubicin liposomes (PEG-LD) and Nab-paclitaxel by increasing tumor vascularization and thus the intratumoral concentration of nanotherapeutic particles.

## RESULTS

### Pegylated doxorubicin liposomes improve the therapeutic response compared to free doxorubicin

**Figure 1:**
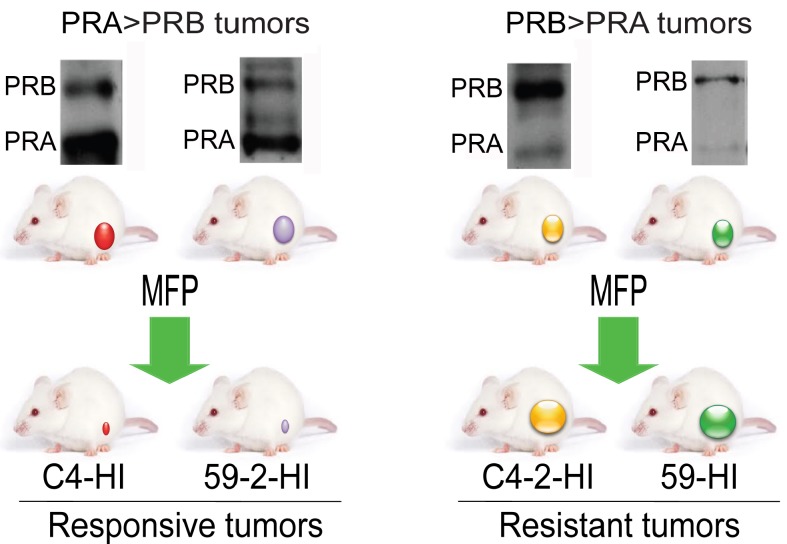
Tumors from the MPA-induced breast cancer model used in this study Hormone-dependent mammary carcinomas induced by MPA are maintained by syngeneic passages in MPA-treated or untreated BALB/c mice. Occasionally hormone independent (HI) variants arise. These HI variants show different PR isoform ratios. C4-2-HI and 59-HI express higher levels of PRB than PRA and are resistant to MFP treatment (Western blots in the right panel). C4-HI and 59-2-HI show higher levels of PRA than PRB (Western blots in the left panel) and are MFP responsive tumors. These four tumors were selected for this study. The model is reviewed in Lanari et al., [32].

**Figure 2:**
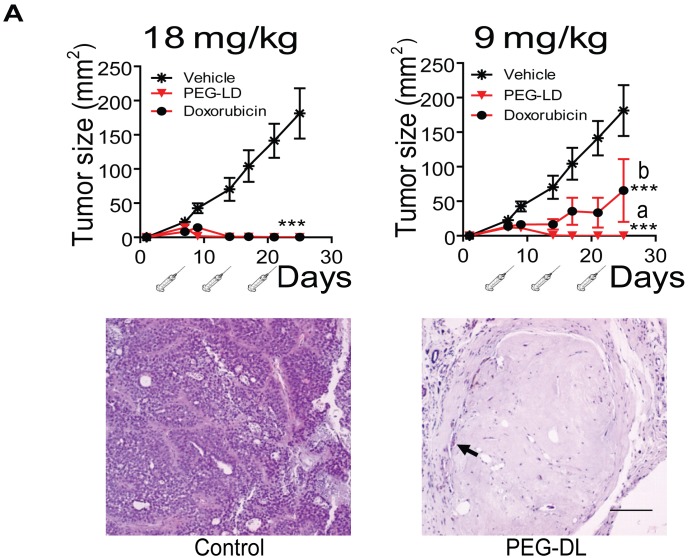
Effect of PEG-LD and free doxorubicin on C4-HI tumor growth Top: Growth curves. Syringes represent injections. With doses of 18 mg/kg, complete tumor regression was observed with both formulations. With doses of 9 mg/kg, PEG-LD showed an improved effect compared with free doxorubicin; a *vs*. b: p<0.05; ***: p<0.001 experimental *vs*. control. Bottom: histological images of tumors treated with 18 mg/kg PEG-LD for one month. Few residual neoplastic cells remained immersed in a dense hyaline stroma (arrow); bar = 100 μm.

**Figure 3:**
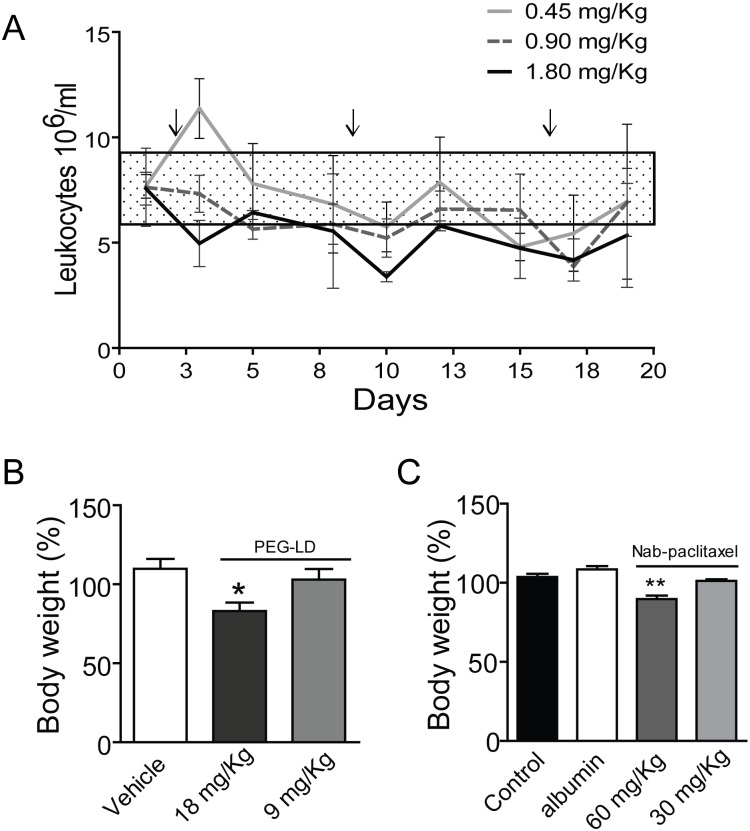
Side effects of PEG-LD and Nab-paclitaxel A. Changes in the body weights of BALB/c female mice after 4 weeks of PEG-DL treatment, with respect to their weights at day 0. Animals received *iv* four weekly doses (n=8). Only mice with the higher dose showed a significant decrease in body weight after treatment. B. Peripheral blood leukocytes in BALB/c mice treated once a week with different low doses of PEG-LD for three weeks. Control values were obtained before treatment initiation. Only the dose of 1.8 mg/kg induced a significant decrease (p<0.05) in the number of leukocytes/ml of blood. The rectangle represents the mean ± SD values observed in untreated mice. C. Nude mice were treated *iv* with three doses of Nab-paclitaxel, albumin or vehicle every four days. Animals were weighted at day 10 after treatment initiation. Only the dose of 60 mg/kg induced a decrease in body weight. **: p<0.01 and p<0.05 experimental *vs*. control group.

We first compared the therapeutic effect of PEG-LD and free doxorubicin using MFP-responsive tumors of the medroxyprogesterone acetate (MPA)-induced breast cancer model (Figure 1). As shown in Figure 2 (top), PEG-LD at 18 and 9 mg/kg induced almost complete tumor regression and was more efficacious as compared with free doxorubicin as observed with the lower dose (p<0.05). Few tumor cells immersed in a dense stromal tissue were observed in PEG-LD treated tumors at the end of the experiment (Figure 2, bottom). However, with the highest dose (18 mg/kg), there were signs of toxicity, such as loss of body weight (Figure 3A) and palmoplantar erythrodysesthesia (not shown). Histologically, these PEG-LD-treated mice had specific skin lesions, with marked acanthopapillomatosis, hyperkeratosis and dermal fibrosis. We also observed mild cardiac hypertrophy. The other histological signs encountered were those typical of a cachectic state. The 9 mg/kg doses showed in a less degree some of the previously mentioned lesions. No other signs were found in mice receiving lower doses during the course of the experiments. These experiments indicated that even lower doses of PEG-LD should be selected for testing in combined treatment experiments.

### MFP improved the therapeutic effect of PEG-LD in murine mammary carcinomas with higher levels of PRA than PRB

**Figure 4:**
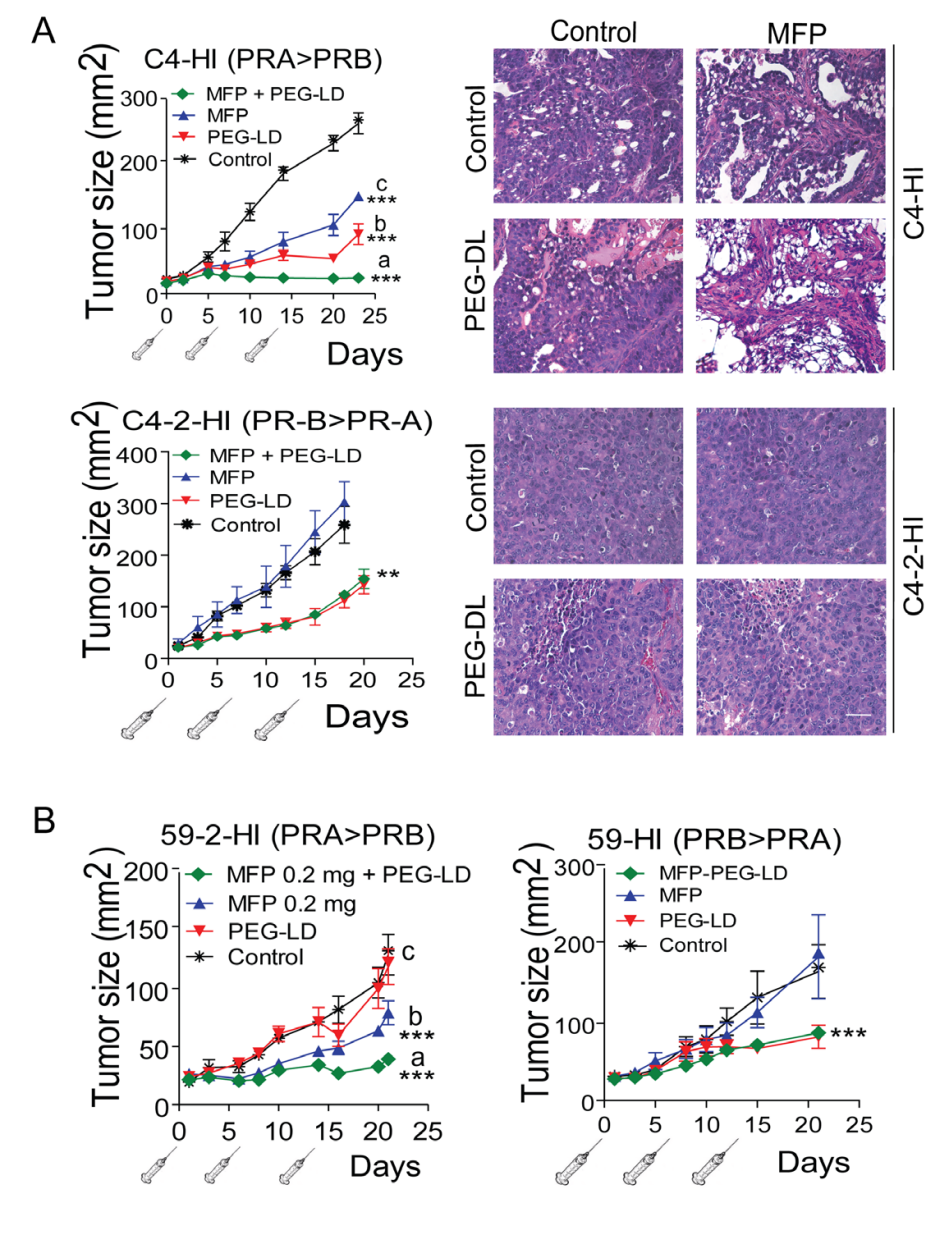
MFP improves the therapeutic effects of PEG-LD only in mammary carcinomas showing higher levels of PRA than PRB A. left: C4-HI (top) or C4-2-HI (bottom) tumors were treated with PEG-LD (0.9 mg/kg) and/or MFP as described in Materials and Methods. An improved therapeutic effect was observed with combined treatments only in C4-HI tumors. Right, histological images of tumors excised after 28 days of treatment show that increased signs of tumor regression were observed with combined treatments only in C4-HI tumors; bar = 40 μm; ***: p<0.001 experimental *vs*. control; a *vs*. b or c: p<0.001. B. 59-2-HI (left) or 59-HI (right) tumors were treated with PEG-LD and/or MFP as described in Materials and Methods. An improved therapeutic effect was observed with combined treatments only in 59-2-HI tumors; ***: p<0.001 experimental *vs*. control; a *vs*. b or c: p<0.001.

We then evaluated the combination of low doses of PEG-LD (Doxopeg; 0.9 or 0.45 mg/kg) together with MFP in two MFP-responsive tumors expressing higher levels of PRA than PRB and in two MFP-resistant tumors with the opposite PR ratio (Figure 1). The doses of PEG-LD and MFP were fixed for each tumor to avoid complete tumor regressions. As shown in Figure 4A and 4B, the combined treatments showed improved effects compared with the single treatments only in MFP-responsive tumors (C4-HI and 59-2-HI). Histological images of C4-HI tumors at the end of the experiment are shown in Figure 4A (upper right panels). MFP induced differentiation and an increase in stromal tissue, as previously reported [28], while PEG-LD increased necrosis. Few tumor cells surrounded by collagen fibers were observed with the combined treatments. In 59-2-HI, MFP induces an increase in the stroma/parenchyma ratio, decreases mitosis and increases apoptosis [30]. This tumor is so sensitive to MFP that we had to reduce the doses as much as 30 times as compared with that used for C4-HI tumors. PEG-LD at very low doses also relatively increased the stromal tissue, and epithelial cells with large bizarre nuclei were observed. Similar images with increased necrotic areas were seen in tumors with combined treatments (not shown).

C4-2-HI (Figure 4A lower panel) and 59-HI (Figure 4B, right) PEG-LD-treated tumors, in which PRB levels were higher than PRA, size and morphology were similar to those of combined treatments. The number of leukocytes remained within the control levels at these low PEG-LD doses (Figure 3B).

### MFP improved the therapeutic effect of Nab-paclitaxel in murine mammary carcinomas with higher levels of PRA than PRB

**Figure 5:**
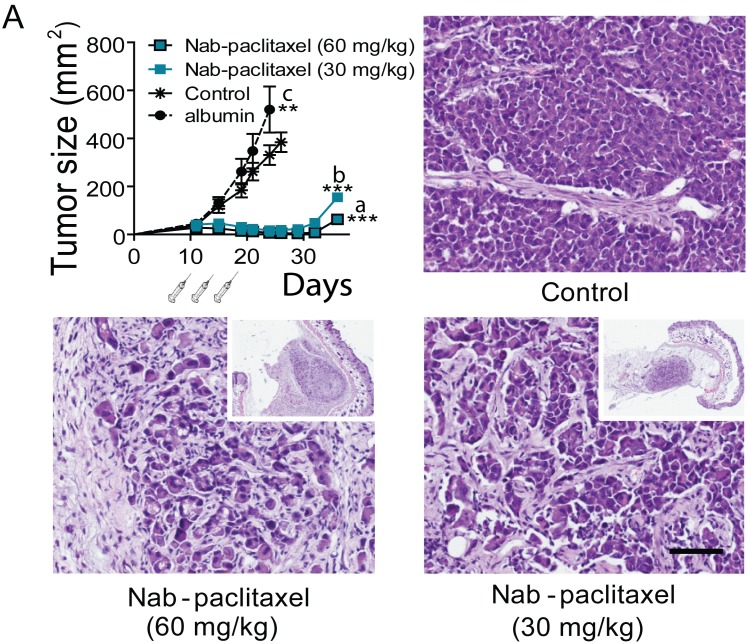
Effect of Nab-paclitaxel on tumor growth C4-2-HI tumors were transplanted into nude mice and treated with 60 or 30 mg/kg Nab-paclitaxel as described in Materials and Methods. Tumors regressed with both Nab-paclitaxel doses. Albumin alone increased tumor growth. The histological images of tumors 28 days after treatment initiation are shown. Control tumors are composed by undifferentiated cells growing as solid sheets. Treated tumors show remaining tumor nests surrounded by stromal tissue; bar = 100 μm. Tumors regressed almost completely with both Nab-paclitaxel doses, and started to grow again 10 days after the last Nab-paclitaxel dose. ***: p<0.001 experimental *vs*. both controls; p<0.05 control group *vs*. control with albumin.

We first tested the effect of three doses of 60 and 30 mg/kg of Nab-paclitaxel on body weight and tumor growth using tumors transplanted in nude mice (to avoid the potential immunogenic effect of the human albumin present in Nab-paclitaxel). Both doses induced a similar inhibitory effect on C4-2-HI tumor growth (Figure 5). Tumors disappeared almost completely after treatment, although they started growing back 10 days after the last dose. Only the dose of 60 mg/kg did induce a significant decrease in body weight (Figure 3C). The animals went back to their original weight within one week after the last dose of Nab-paclitaxel, and no specific histological alterations were found in autopsies of this group.

**Figure 6:**
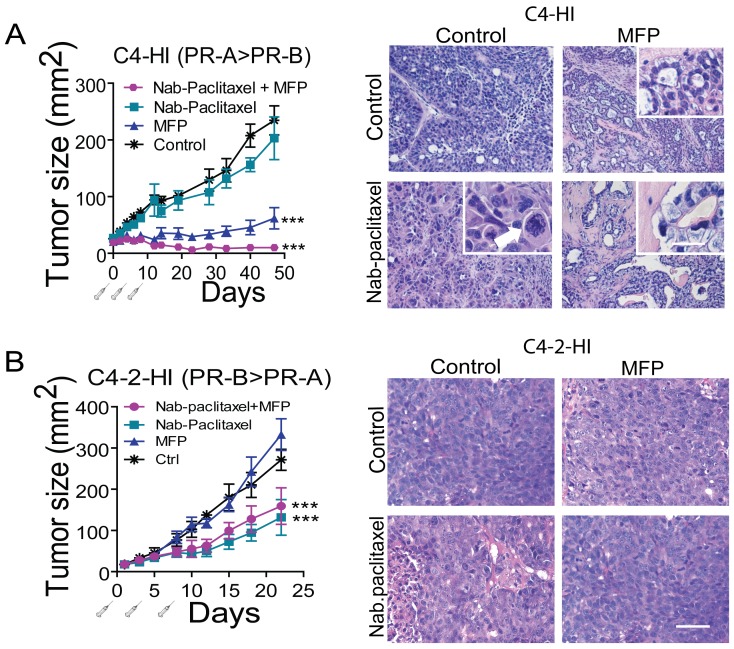
MFP improves the therapeutic effects of Nab-paclitaxel only in mammary carcinomas showing higher levels of PRA than PRB A. left: nude mice with palpable C4-HI tumors were treated as explained in Materials and Methods with Nab-paclitaxel (30 mg/kg), MFP, or both treatments (n=6/group). MFP induced an inhibitory effect on tumor growth that was greater when combined with Nab-paclitaxel. ***: p<0.001 experimental *vs*. control; a *vs*. b or c: p<0.001. Right: Representative images of tumors excised 10 days after treatment initiation. Nab-paclitaxel induced an early increase in aberrant cells with multinucleated pleomorphic nuclei showing partially condensed chromatin. The inset shows a tetrapolar mitosis (arrow head) and a mitotic cell with chromosomal spreading (arrow). Eosinophilic cells and cells with large amounts of cytoplasm were also encountered. MFP induced differentiation. In (Nab-paclitaxel+MFP)-treated tumors, the combined treatment mimics the effect of antiprogestin, although a higher number of aberrant signet ring-like cells can be observed (inset). B. left: Nab-paclitaxel (15 mg/kg) inhibited the growth of C4-2-HI, and MFP did not improve the effect of Nab-paclitaxel. Right: No morphological differences were observed in C4-2-HI tumors with the combined treatments. Bar = 40 μm; inset bar = 20 μm.

We then studied the effect of Nab-paclitaxel (30 mg/kg) on the growth of antiprogestin-responsive C4-HI tumors. This dose did not significantly inhibit tumor growth. However, co-treatment with MFP improved the inhibitory effect induced by MFP (Figure 6A left). Selected animals were euthanized 10 days after treatment initiation). Nab-paclitaxel treatment induced aberrant mitotic figures and multiple hyperchromic nuclei (Figure 6A right). However, tumor histoarchitecture was essentially similar to that of the control tumors, and the tumors continued growing. MFP induced very early tumor differentiation, as expected [28], and MFP + Nab-paclitaxel-treated tumors showed a differentiation pattern similar to that of MFP-treated tumors. The Nab-paclitaxel signature was usually present shortly after treatment began, and giant cells with dense chromatin were frequently seen (Figure 6A, right). At the end of treatment, in most cases, more than 90 % of the tumor cells were lost (not shown). No additive effect was found in C4-2-HI tumors co-treated with Nab-paclitaxel (15 mg/kg) and MFP (Figure 6B).

### MFP improved the therapeutic effect of both PEG-LD and Nab-paclitaxel in T47D-YA but not in T47D-YB xenografts growing in NSG mice

**Figure 7:**
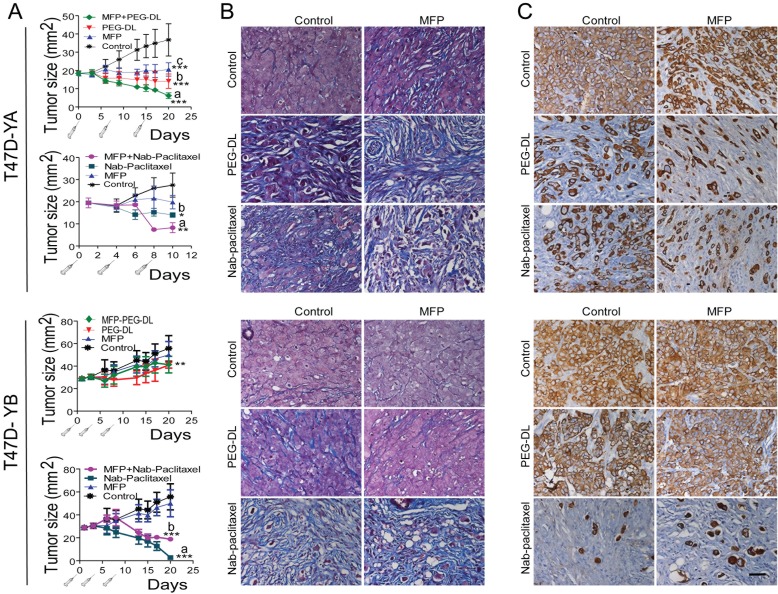
MFP improves the therapeutic effects of PEG-LD and Nab-paclitaxel only in xenografts overexpressing PRA A. T47D-YA or T47D-YB xenografts growing with E2 in NSG mice (n=6/group) were treated with PEG-LD (0.9 mg/kg) or Nab-paclitaxel (15 mg/kg) and/or MFP (10 mg/kg/day). MFP only inhibited the growth of T47D-YA xenografts; PEG-LD was more effective in T47D-YA tumors compared to T47D-YB tumors, and the latter were more sensitive to Nab-paclitaxel than T47D-YA tumors. Improved effects with combined treatments were only observed in the T47D-YA tumors. ***: p<0.001; **: p<0.01; and *: p<0.05 (experimental *vs*. control). T47D-YA, top a *vs*. b: p<0.05; a *vs*. c: p<0.001; bottom: a *vs*. b or c: p<0.05. T47D-YB, bottom: a *vs*. b: p<0.05; a *vs*. c: p<0.001. B. Masson's trichrome staining. High levels of collagen (blue fibers) can be observed in all regressing tumors. C. CK staining. The amount of epithelial cells (CK+) in relation to the total number of cells can be observed. Few epithelial tumor cells remain after combined treatments in T47D-YA tumors. Conversely, for T47D-YB, the best therapeutic option was Nab-paclitaxel. Bar = 40 μm.

**Figure 8:**
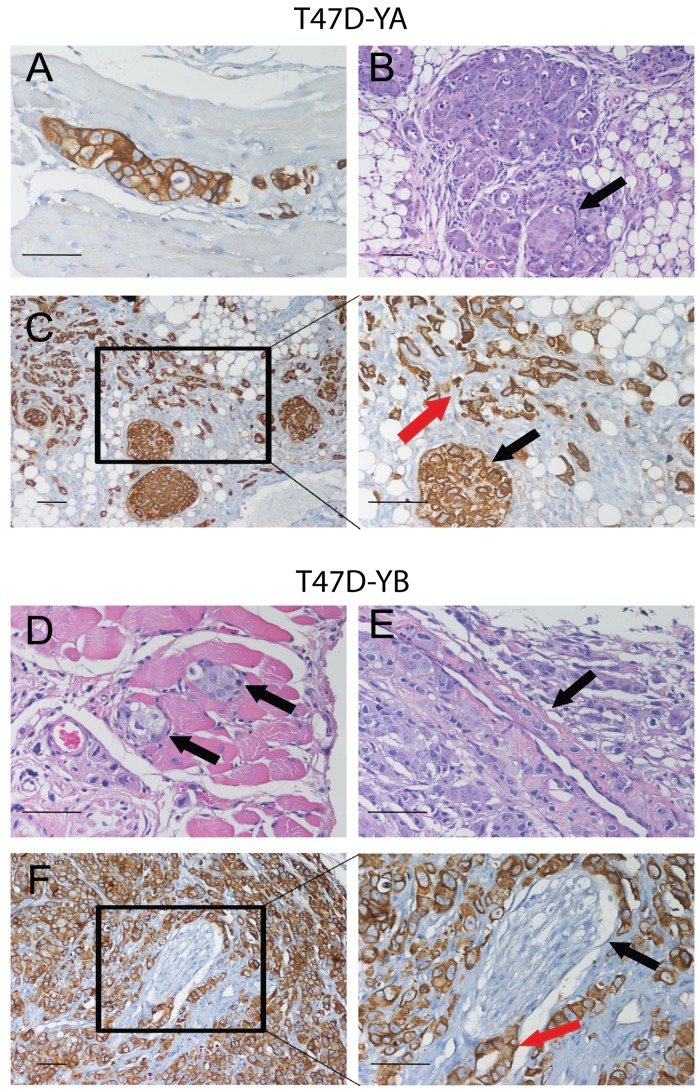
Images of T47D-YA and T47D-YB cells growing in vessels, muscle or nerve fibers, or inside the mammary gland ducts Top panel: T47D-YA tumor cells (CK+) invading muscle fibers (A), or growing inside an alveolar structure of the mammary gland (B; H&E), C. Images of PEG-LD treated-tumors. Red arrow shows small groups of remaining CK+ tumor cells surrounded by stromal tissue. Black arrow shows tumor cells growing inside a ductal structure. Bottom panel: T47D-YB tumor cells growing in between the muscle skeletal fibers (D), or lining the wall of a vessel (E). F. Perineural invasion of cells growing surrounding a nerve fiber. Black arrow: nerve sheath. Red arrow: tumor cells in the perineural space. An antibody that only recognizes human CK was used. Bar = 20 μm.

MFP inhibited the growth of PRA-overexpressing xenografts, as expected (Wargon and Riggio et al, submitted). Nab-paclitaxel had an increased inhibitory effect on T47D-YB xenografts, while PEG-LD had it on T47D-YA tumors. We observed an improved therapeutic effect with combined treatments only in T47D-YA tumors (Figure 7A). Moreover, MFP lowered the inhibitory effect of Nab-paclitaxel in the T47D-YB tumors. Large amounts of collagen fibers were observed in all regressing tumors (Masson's trichrome staining; Figure 7B). Cytokeratin (CK) staining (Figure 7C) revealed the relative amount of epithelial tumor cells in relation to the tumor mass, indicating that the therapeutic effect was even greater than expected based on the values obtained by measuring the tumors. Interestingly, T47D cells grow invading muscle, nerves or grow inside the mammary gland ducts, and the latter were protected from the chemotherapeutic effects of the drugs (Figure 8).

### MFP modifies the microenvironment and microvasculature of responsive tumors, inducing an increase in nanoparticle accumulation

**Figure 9:**
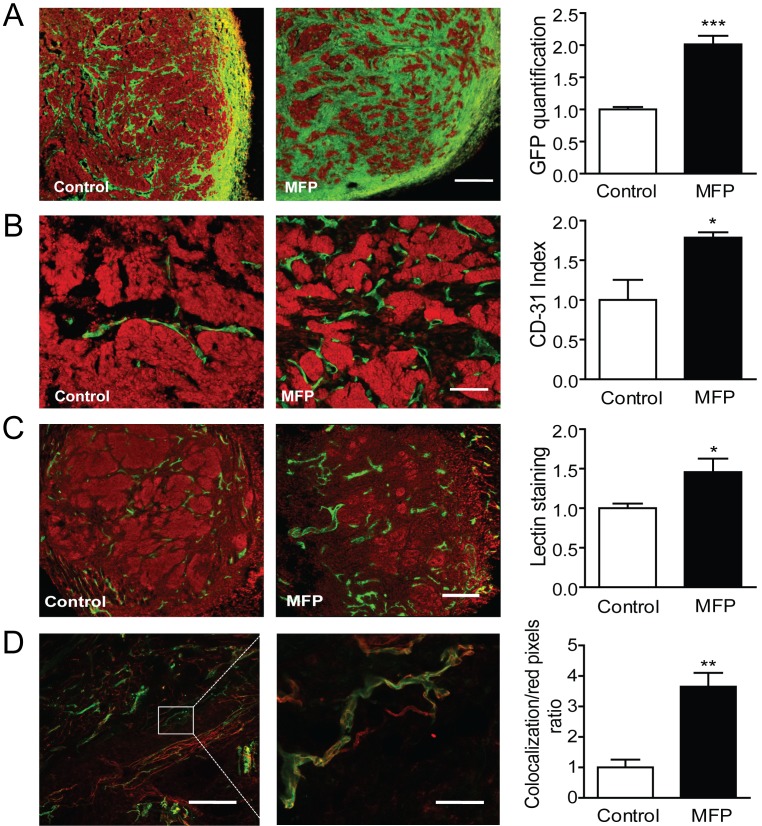
MFP treatment increased tumor remodeling and the amount of functional vessels in mammary carcinomas showing higher levels of PRA than PRB A. 59-2-HI tumors growing in BALB/c-GFP mice were treated or not for 5 days with MFP (10 mg/kg/day). An increase in stromal cells (green cells; GFP) was observed in MFP-treated tumors. Nuclei were counterstained with PI (red); bar = 300 µm. B. Similar experiments with tumors growing in BALB/c mice. An increase in CD31 positive cells (green staining; FITC) was observed in MFP-treated tumors. Nuclei were counterstained with PI; bar = 300 µm. C. TL were *iv* injected in mice bearing tumors treated as described in B. Mice were euthanized and tumors processed for IF studies. PI was used for nuclear staining. Increased total lectin fluorescence (green) was observed in MFP-treated tumors; bar = 300 µm. D. Co-localization between TL (green) and CD31 immunostaining (red). Increased co-localization was observed in MFP-treated tumors (yellow); left bar = 300 µm; right bar = 50 µm.

C4-HI regresses under antiprogestin treatment by increasing differentiation and stromal tissue production (Figure 6A top), and 59-2-HI regresses by increasing the stroma/parenchyma ratio (Figure 9A). Using the 59-2-HI tumor, we explored whether MFP induced a change in the number of vessels. An increase in the number of CD31-positive cells (endothelial cells) was observed in MFP-treated tumors (Figure 9B). In addition, fluorescent tomato lectin (TL), which binds to endothelial cells, was injected *in vivo* as described in Materials and Methods, and an increase in staining was observed in MFP-treated tumors (Figure 9C). Co-localization between CD31 and TL confirmed the increase in vessel functionality in MFP-treated tumors (Figure 9D).

**Figure 10:**
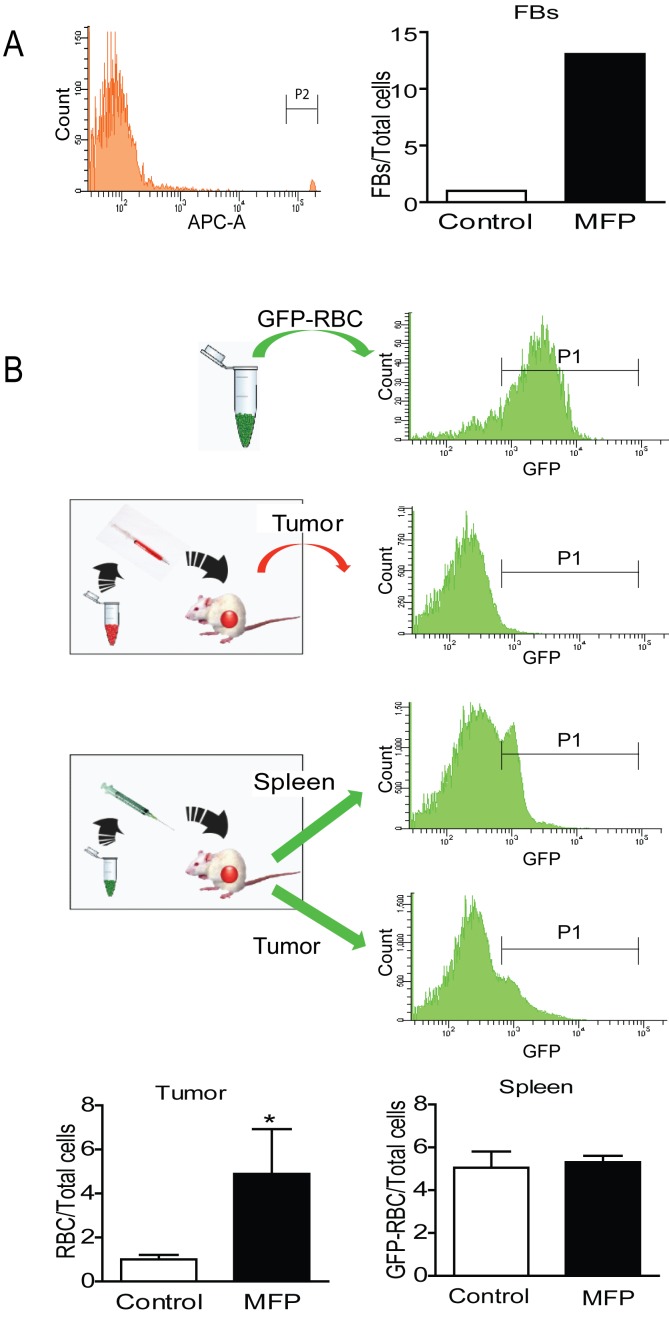
MFP increased the amounts of FB or GFP-RBC in tumors with higher levels of PRA than PRB A. FACS analysis of FB injected *iv* in MFP-treated tumors and untreated mice bearing 59-2-HI tumors. Sixty minutes later mice were euthanized and tumors processed for FACS analysis. An increase in FB was observed in MFP-treated tumors. B, FACS analysis of GFPRBC injected as in A. GFP-RBC were prepared as described in Materials and Methods and were inoculated *iv* in MFP-treated or untreated tumor bearing mice. Top panel: FACS analysis of GFP-RBC (positive control) and middle panel negative control. Bottom panels: the content of GFP-RBC was evaluated after 3 days by FACS analysis in tumor tissue or in spleens. An increase of GFP-RBC was observed in tumors and not in spleens from MFP-treated mice as compared with untreated mice. *: p<0.05; **: p<0.01 and ***: p<0.001. Frequency corresponds to the number of GFP-RCB (P1) gated on the erythrocyte population in the FAC-A vs. the SSC-A dot plot.

To evaluate whether this increased vascularization could be responsible for an increase in drug accumulation, count control Fluorescent Beads for Flow Cytometry** (FB) or purified red blood cells (RBC) obtained from transgenic mice expressing enhanced Green Fluorescent Protein (GFP) under the direction of the human ubiquitin C promoter (GFP-RBC) were injected *iv* into mice bearing tumors that were treated or not with MFP, and their presence inside the tumors was quantified by FACS. An increase in FB (Figure 10A) or GFP-RBC (Figure 10B and 10C) was found in the tumors from MFP-treated mice, while no differences were found in the GFP-RBC content in their spleens (Figure 10B).

**Figure 11:**
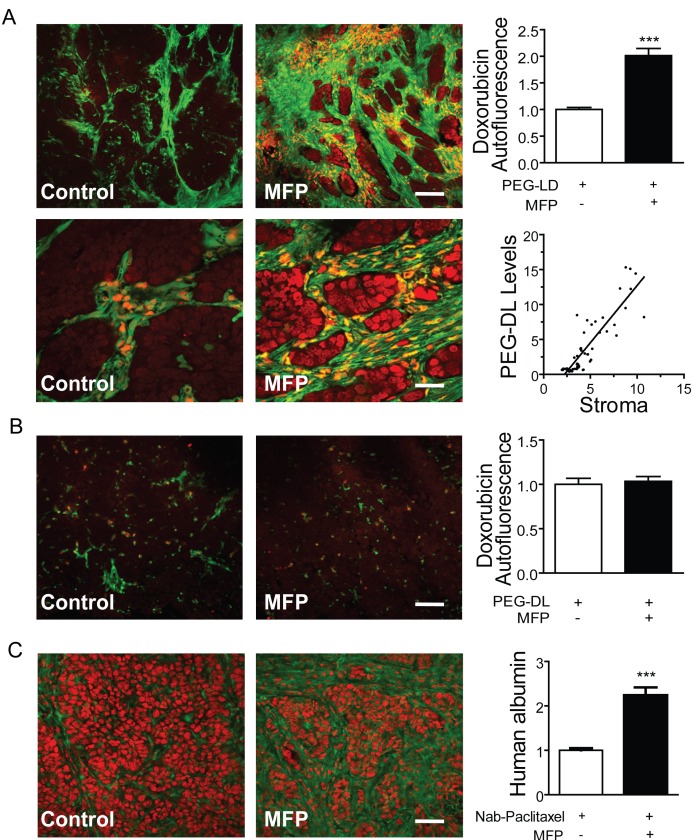
MFP increased the amounts of PEG-LD or Nab-paclitaxel in tumors with higher levels of PRA than PRB A. 59-2-HI tumors were transplanted in BALB/c-GFP mice and were treated or not with MFP as described in Figure 9. PEG-LD (18 mg/kg) were injected *iv* and after 24 h tumors were excised and processed for confocal microscopy studies. The red staining corresponds to the doxorubicin autofluorescence. Notice the red staining in stromal cells (orange; fill arrow) and in epithelial tumor cells nearby the stromal cells; top bar = 120 μm, bottom bar = 30 μm; B. C4-2-HI tumors were similarly treated and no differences in doxorubicin content were observed between treated and untreated tumors; bar = 120 μm. C. Tumors growing in nude mice were treated with MFP as described above and Nab-paclitaxel (60 mg/kg) was injected *iv*. After 24 h tumors were excised and processed for IF studies. An anti human albumin antibody and a FITC-coupled secondary antibody (green) were used. PI was used for nuclear counterstaining. No staining was observed in negative controls (treated without primary antibodies or in untreated tumors); bar = 120 μm *: p<0.05; **: p<0.01 and ***: p<0.001.

Next, we took advantage of doxorubicin's red autofluorescence. Tumors growing in BALB/c-GFP mice were treated as described in Materials and Methods. As shown in Figure 11A, red nuclear staining was observed in stromal cells and in the tumor cells adjacent to them (green cells). The total amount of doxorubicin was much higher in the tumors subjected to the combined treatments. No differences were observed in similarly treated C4-2-HI tumors (Figure 11B).

Similarly, the amount of Nab-paclitaxel present in the tumors was indirectly evaluated using a polyclonal anti-human albumin antibody. Increased staining was found in animals subjected to combined treatments (Figure 11C). No staining was observed in the negative controls.

## DISCUSSION

In this study we show that mammary carcinomas expressing different ratios of PR isoforms are sensitive to both PEG-LD (anthracycline) and Nab-paclitaxel (taxane). Antiprogestin-resistant tumors were very sensitive to Nab-paclitaxel, whereas tumors with higher levels of PRA than PRB were less sensitive (C4-HI vs. C4-2-HI and T47D-YA vs. T47D-YB). The opposite result was observed with PEG-LD treatment. Targeting PRA-overexpressing tumors with antiprogestins resensitized the tumors to both chemotherapeutic treatments.

Because chemotherapy is the therapy of choice for metastatic breast disease, an ideal scenario would be to improve its efficacy by specifically increasing the delivery into tumor cells, thus reducing potentially deleterious side effects associated with normal tissue targeting. Nab-paclitaxel accumulates in cancer cells due to their increased ability to engulf albumin [32]. Pegylated liposomes are also concentrated in tumor cells because of the leaky tumor vasculature [16]. Antiprogestin treatment may increase the efficiency of these highly selective agents in a subgroup of breast cancer patients: those overexpressing PRA.

To explain the potential mechanisms involved in this increased efficacy, we examined several possibilities. It has been reported that MFP and progesterone are able to bind multidrug-resistant proteins and thus improve the entry of chemotherapeutic agents into the cells [33;34]. Although this may be a very interesting possibility to explore, it does not seem to be the prevailing mechanism because almost complete tumor regressions were observed with anthracyclines in this model (Figure 2A), which is a response that is not compatible with a multidrug resistance mechanism. Therefore, we explored the possibility that the tissue remodeling induced by antiprogestin treatment could be responsible for the drug accumulation. Accordingly, it has been demonstrated that the therapeutic effects of doxorubicin were decreased in KO mice lacking MMP-9 [35], and interestingly, MMP-9 expression is increased in tumors of the MPA breast cancer model treated with MFP [31].

Using endothelial markers, we showed an increase in the number of capillary-type vessels within the tumor. This result, although counterintuitive at first, also negates the idea that diminished angiogenesis might play a role in MFP-induced tumor regression. Conversely, these new functional vessels participate in the distribution of the chemotherapeutic drugs within the tumor. A proangiogenic effect may be the direct outcome of an initial hypoxic state induced by the first dose of the chemotherapeutic agent. However, since MFP alone induces apoptosis, necrosis and/or cytostasis in the tumor parenchyma of PRA-overexpressing tumors, it seems likely that this increased angiogenesis is driven by non-specific stromal changes, secondary to epithelial cell death. The mechanisms that trigger these effects deserve further investigation. Our results are in agreement with those reported by Nakasone *et al*., who found that free doxorubicin accumulates in cells flanking tumor vessels in two different transgenic mouse models [35]. In support of the remodeling hypothesis, we also demonstrated that estrogen treatment, which also induces tumor regression in this model [36], is also able to increase the therapeutic effect of these chemotherapeutic drugs (data not shown). Other drugs have also been proposed to alter the tumor microenvironment such as hialuronidase that improved the access of PEG-LD in osteosarcoma xenografts [37], or losartan, an angiotensin receptor inhibitor that inhibits the synthesis of collagen type I present in desmoplastic tumors [38]. Losartan improved the therapeutic effect of 4 mg/kg PEG-LD, which is a suboptimal chemotherapeutic dose that is 4 times higher than the doses used in our assays. The doses of PEG-LD used is our study ranged from 18 mg/kg (equivalent to a human dose of 54 mg/m^2^) to 0.45 mg/kg (equivalent to 1.85 mg/m^2^)[39]. For breast cancer treatment a dose of 50 mg/m^2^ is recommended.

Our studies also suggest that anthracyclines may be a better option than taxanes for the treatment of tumors overexpressing PRA, while taxanes may be better for those expressing PRB. The reason for this selectivity is still not known. For paclitaxel, it can be speculated that PRA-overexpressing tumors have increased levels of BCL-XL or BCL-2 than their PRB counterparts [40]; alternatively, they may have increased levels of other proteins related to microtubule assembly that are oppositely regulated by taxanes [26]. An excess of these proteins may be decreasing paclitaxel efficacy.

Some of the histopathological observations regarding the paclitaxel effect may deserve further investigation. We are currently exploring the biological consequences that the presence of aberrant mitosis may have in tumors that are not growth inhibited by paclitaxel at doses of 30 mg/kg such as C4-HI. These observations may have clinical relevance, as they indicate that paclitaxel treatment should be potent enough to destroy the majority of proliferating cells, thus avoiding the selection of more aggressive phenotypes. In the same line, it has been recently reported that paclitaxel may increase polyploidy and therefore alter the status of erbB2 expression [41].

There are several reports regarding the effect of combined chemotherapeutic treatments and endocrine therapies. In preclinical studies involving fulvestrant or tamoxifen, it has been shown that fulvestrant exerts synergic effects with other chemotherapeutic agents under experimental conditions in which tamoxifen failed [42]. In clinical studies in which combined or sequential adjuvant tamoxifen and chemotherapy were compared, no beneficial effects were observed with combined treatments [43]. Hypotheses predicting an additive or synergistic effect, such as reversal of p170-mediated resistance to anthracyclines by tamoxifen, appeared not to play a clinically relevant role. The most accepted explanation for this failure was that tamoxifen decreased the number of proliferating cells, thus creating cytokinetic resistance to chemotherapy. It can be hypothesized that in the absence of a significant tumor mass, as occurs in adjuvant therapies, the remodeling effect of endocrine therapy described in this study does not take place. Accordingly, the mechanisms we describe here may synergize with those of chemotherapeutic agents delivered as nanoparticles in combination with an endocrine therapy, while lowering potential side effects. We expect that in the near future, antiprogestins might be an option for breast cancer patients overexpressing PRA.

Interestingly, Skor *et al* [44] reported that MFP increased the efficiency of paclitaxel in triple negative xenografts, thus counteracting the protective effect that glucocorticoid receptors (GR) exert on chemotherapy. Because these results indicate a beneficial effect of MFP in combination with paclitaxel, it may be argued that GR might also be playing a similar role in our studies. However, the fact that T47D-YA and T47D-YB xenografts only differ in the introduced PR isoform and that only the T47D-YA xenografts showed improvement with combined therapies suggests that this is not the prevailing mechanism.

In summary, we propose that breast cancer patients with advanced disease should be categorized according to the prevailing PR isoform expressed. For those expressing high levels of PRA, the combination of an antiprogestin together with a chemotherapeutic agent may allow for the use of lower chemotherapeutic doses, mainly in neo-adjuvant protocols, thus improving the quality of life.

## MATERIALS AND METHODS

### Animals

Two-month-old virgin female BALB/c mice (Animal Facility, IBYME) were used. Transgenic mice expressing enhanced Green Fluorescent Protein (GFP) under the direction of the human ubiquitin C promoter (The Jackson Laboratories, Bar Harbor, Maine) were bred at IBYME (BALB/c-GFP). Nu/nu females were obtained from the University of La Plata and NOD/LtSz-scid/IL-2Rgamma null mice (NSG) from The Jackson Laboratory and bred at IBYME. Animal care and manipulation were in agreement with the Guide for the Care and Use of Laboratory Animals [45].

### Tumors and xenografts

C4-HI, C4-2-HI, 59-HI or 59-2-HI carcinomas from the MPA-induced murine breast cancer model [46], all expressing ER and PR, were orthotopically transplanted into the mammary gland 4 of BALB/c, BALB/c-GFP or nu/nu mice. C4-HI and 59-2-HI tumors regress with MFP treatment and express levels of PRA higher than those of PRB; C4-2-HI and 59-HI are constitutive MFP-resistant tumor variants with levels of PRB higher than those of PRA (Figure 1) [28]. T47D-YA and T47D-YB cells were a kind gift of Dr. K. Horwitz (University of Colorado) and were cultured as previously described [47]. Cells (5×10^6^) were inoculated orthotopically into NSG female mice. One week prior to cell inoculation, E_2_ silastic pellets containing 0.5 mg 17-βestraciol (E_2_) were subcutaneously (sc) implanted [48].

### Treatments

Free doxorubicin and the PEG-LD Doxopeg were a kind gift of Laboratorios Raffo (Buenos Aires) whereas Nab-paclitaxel Abraxane (Abraxis Bioscience) is a commercial preparation. PEG-LD vehicle and free human albumin, provided by Laboratorios Raffo, were used as a control in PEG-LD or Nab-paclitaxel experiments respectively. PEG-LD or doxorubicin were administered intravenously (iv) in a range of 18-0.45 mg/kg body weight once a week. For the highest dose we used a protocol adapted from Charrois and Allen [49]. Three consecutive doses of 60, 30 or 15 mg/kg of Nab-paclitaxel were given iv every 4 days [50]. MFP (Sigma, St Louis, MI) was administered using 6 or 0.2 mg pellets implanted subcutaneously (sc) or by sc daily injections of 10 mg/kg body weight. Tumor size was evaluated using a Vernier caliper. Mice were euthanized, and transplanted tumors and metastases were fixed in buffered formalin and embedded in paraffin. Five microns sections were used for histological diagnoses (H-E staining and Masson trichrome). The pathological response to treatments was assessed according to Miller and Payne grading system [51]. To evaluate the intra tumor drug content, mice were treated for 5 days with MFP, and then with Nab-albumin or PEG-LD for 24 hours.

### Side effects

Mice without tumors (6-8 per group) were treated as described above, and weighed once a week (PEG-LD) or every four days (Nab-paclitaxel). PEG-LD-treated mice were sacrificed after one month and Nab-paclitaxel-treated mice after 10 days of treatment. For leukocyte counting, PEG-LD was administered once a week and mice were bled 3 times a week during 20 days (n=8). Leukocytes were counted using the analyzer A^c^ T diff of Coulter.

### Immunofluorescence

Frozen tumor sections (15 µm) were fixed in 10% formalin, transferred to 70% ethanol, blocked, and successively incubated overnight with CD31 (550274, BD Pharmingen) or with anti human albumin (ab2406; Abcam), antibodies and processed as described previously (32). The nuclei were counterstained with propidium iodide (PI) and analyzed using a Nikon Eclipse E800 confocal Microscope. Images were taken with a Nikon DS-U1 camera with ACT-2U software. BALB/c-GFP mice were perfused with a cold saline solution (0.9% NaCl) followed by 4% paraformaldehyde before tumor excision. Excised tumors were kept in cold 4% paraformaldehyde overnight and transferred to 20% sucrose for another 24 hours. Endogenous doxorubicin or GFP fluorescence were analyzed by confocal microscopy.

### Immunohistochemistry

It was carried out as described previously [30] using the CK antibody from Dako (clone AE1/AE3).Quantification of Stroma, TL, Count control Fluorescent beads for Flow Cytometry (FB) or GFP-red blood cells (RBC)TL (70 µl of a 20 mg/ml solution; Vector Labs, Burlingame, CA), FB (3.5 × 10^6^; S236630, Dako) or purified GFP-RBC (6 × 10^6^) [52] were injected iv into MFP-treated or untreated mice bearing tumors. After 7 (TL) or 60 minutes (FB), or 72 hours (RBC), the mice were euthanized and tumors processed for confocal microscopy (TL) or flow cytometry (FB and GFP-RBC).

### Statistical analysis

GraphPad Prism (version 5.0) was used for statistical analysis. ANOVA and Bonferroni multiple post t tests were used to evaluate differences of means of multiple samples, and Student's t test of two different groups. Tumor growth curves were studied using regression analysis and slopes compared using one-way ANOVA followed by parallelism analysis. In all graphs, the mean ± SEM is shown. Experiments were repeated at least twice.

## References

[1] Rowinsky EK, Donehower RC (1995). Paclitaxel (taxol). N.Engl.J.Med.

[2] (1998). Polychemotherapy for early breast cancer: an overview of the randomised trials. Early Breast Cancer Trialists' Collaborative Group. Lancet.

[3] Wani MC, Taylor HL, Wall ME, Coggon P, McPhail AT (1971). Plant antitumor agents. VI. The isolation and structure of taxol, a novel antileukemic and antitumor agent from Taxus brevifolia. J.Am.Chem.Soc.

[4] Schiff PB, Fant J, Horwitz SB (1979). Promotion of microtubule assembly in vitro by taxol. Nature.

[5] Schiff PB, Horwitz SB (1980). Taxol stabilizes microtubules in mouse fibroblast cells. Proc.Natl.Acad.Sci.U.S.A.

[6] Srivastava RK, Srivastava AR, Korsmeyer SJ, Nesterova M, Cho-Chung YS, Longo DL (1998). Involvement of microtubules in the regulation of Bcl2 phosphorylation and apoptosis through cyclic AMP-dependent protein kinase. Mol. Cell Biol.

[7] Chadebech P, Brichese L, Baldin V, Vidal S, Valette A (1999). Phosphorylation and proteasome-dependent degradation of Bcl-2 in mitotic-arrested cells after microtubule damage. Biochem.Biophys.Res Commun.

[8] Adams JD, Flora KP, Goldspiel BR, Wilson JW, Arbuck SG, Finley R (1993). Taxol: a history of pharmaceutical development and current pharmaceutical concerns. J.Natl.Cancer Inst.Monogr.

[9] Riondel J, Jacrot M, Picot F, Beriel H, Mouriquand C, Potier P (1986). Therapeutic response to taxol of six human tumors xenografted into nude mice. Cancer Chemother. Pharmacol.

[10] Rowinsky EK, Cazenave LA, Donehower RC (1990). Taxol: a novel investigational antimicrotubule agent. J.Natl.Cancer Inst.

[11] Dye D, Watkins J (1980). Suspected anaphylactic reaction to Cremophor EL. Br.Med.J.

[12] Gelderblom H, Verweij J, Nooter K, Sparreboom A (2001). Cremophor EL: the drawbacks and advantages of vehicle selection for drug formulation. Eur.J.Cancer.

[13] Ibrahim NK, Desai N, Legha S, Soon-Shiong P, Theriault RL, Rivera E, Esmaeli B, Ring SE, Bedikian A, Hortobagyi GN, Ellerhorst JA (2002). Phase I and pharmacokinetic study of ABI-007, a Cremophor-free, protein-stabilized, nanoparticle formulation of paclitaxel. Clin.Cancer Res.

[14] Gradishar WJ, Tjulandin S, Davidson N, Shaw H, Desai N, Bhar P, Hawkins M, O'Shaughnessy J (2005). Phase III trial of nanoparticle albumin-bound paclitaxel compared with polyethylated castor oil-based paclitaxel in women with breast cancer. J.Clin.Oncol.

[15] Lomovskaya N, Otten SL, Doi-Katayama Y, Fonstein L, Liu XC, Takatsu T, Inventi-Solari A, Filippini S, Torti F, Colombo AL, Hutchinson CR (1999). Doxorubicin overproduction in Streptomyces peucetius: cloning and characterization of the dnrU ketoreductase and dnrV genes and the doxA cytochrome P-450 hydroxylase gene. J. Bacteriol.

[16] Von Hoff DD, Layard MW, Basa P, Davis HL, Von Hoff AL (1979). Rozencweig M and Muggia FM. Risk factors for doxorubicin-induced congestive heart failure. Ann.Intern.Med.

[17] Symon Z, Peyser A, Tzemach D, Lyass O, Sucher E, Shezen E, Gabizon A (1999). Selective delivery of doxorubicin to patients with breast carcinoma metastases by stealth liposomes. Cancer.

[18] Harris JR, Lippman ME, Veronesi U, Willett W (1992). Breast cancer (1). N.Engl.J.Med.

[19] Lanari C, Wargon V, Rojas P, Molinolo AA (2012). Antiprogestins in breast cancer treatment: are we ready?. Endocr.Relat Cancer.

[20] Liang Y, Besch-Williford C, Brekken RA, Hyder SM (2007). Progestin-dependent progression of human breast tumor xenografts: a novel model for evaluating antitumor therapeutics. Cancer Res.

[21] Graham JD, Yeates C, Balleine RL, Harvey SS, Milliken JS, Bilous AM, Clarke CL (1995). Characterization of progesterone receptor A and B expression in human breast cancer. Cancer Res.

[22] Hopp TA, Weiss HL, Hilsenbeck SG, Cui Y, Allred DC, Horwitz KB, Fuqua SA (2004). Breast cancer patients with progesterone receptor PR-A-rich tumors have poorer disease-free survival rates. Clin. Cancer Res.

[23] Mote PA, Graham JD, Clarke CL (2007). Progesterone receptor isoforms in normal and malignant breast. Ernst.Schering.Found.Symp.Proc.

[24] Bamberger AM, Milde-Langosch K, Schulte HM, Loning T (2000). Progesterone receptor isoforms, PR-B and PR-A, in breast cancer: correlations with clinicopathologic tumor parameters and expression of AP-1 factors. Horm.Res.

[25] Mote PA, Bartow S, Tran N, Clarke CL (2002). Loss of co-ordinate expression of progesterone receptors A and B is an early event in breast carcinogenesis. Breast Cancer Res Treat.

[26] Badtke MM, Jambal P, Dye WW, Spillman MA, Post MD, Horwitz KB, Jacobsen BM (2012). Unliganded progesterone receptors attenuate taxane-induced breast cancer cell death by modulating the spindle assembly checkpoint. Breast Cancer Res Treat.

[27] Helguero LA, Viegas M, Asaithamby A, Shyamala G, Lanari C, Molinolo AA (2003). Progesterone receptor expression in medroxyprogesterone acetate-induced murine mammary carcinomas and response to endocrine treatment. Breast Cancer Res. Treat.

[28] Wargon V, Helguero LA, Bolado J, Rojas P, Novaro V, Molinolo A, Lanari C (2009). Reversal of antiprogestin resistance and progesterone receptor isoform ratio in acquired resistant mammary carcinomas. Breast Cancer Res. Treat.

[29] Vanzulli SI, Soldati R, Meiss R, Colombo L, Molinolo AA, Lanari C (2005). Estrogen or antiprogestin treatment induces complete regression of pulmonary and axillary metastases in an experimental model of breast cancer progression. Carcinogenesis.

[30] Vanzulli S, Efeyan A, Benavides F, Helguero LA, Peters G, Shen J, Conti CJ, Lanari C, Molinolo A (2002). p21, p27 and p53 in estrogen and antiprogestin-induced tumor regression of experimental mouse mammary ductal carcinomas. Carcinogenesis.

[31] Simian M, Molinolo A, Lanari C (2006). Involvement of matrix metalloproteinase activity in hormone-induced mammary tumor regression. Am J Pathol.

[32] Kratz F (2008). Albumin as a drug carrier: design of prodrugs, drug conjugates and nanoparticles. J.Control Release.

[33] Gruol DJ, Zee MC, Trotter J, Bourgeois S (1994). Reversal of multidrug resistance by RU 486. Cancer Res.

[34] Lecureur V, Fardel O, Guillouzo A (1994). The antiprogestatin drug RU 486 potentiates doxorubicin cytotoxicity in multidrug resistant cells through inhibition of P-glycoprotein function. FEBS Lett.

[35] Nakasone ES, Askautrud HA, Kees T, Park JH, Plaks V, Ewald AJ, Fein M, Rasch MG, Tan YX, Qiu J, Park J, Sinha P, Bissell MJ, Frengen E, Werb Z, Egeblad M (2012). Imaging Tumor-Stroma Interactions during Chemotherapy Reveals Contributions of the Microenvironment to Resistance. Cancer Cell.

[36] Soldati R, Wargon V, Cerliani JP, Giulianelli S, Vanzulli SI, Gorostiaga MA, Bolado J, do CP, Molinolo A, Vollmer G, Lanari C (2009). Inhibition of mammary tumor growth by estrogens: is there a specific role for estrogen receptors alpha and beta?. Breast Cancer Res Treat.

[37] Eikenes L, Tari M, Tufto I, Bruland OS, de Lange DC (2005). Hyaluronidase induces a transcapillary pressure gradient and improves the distribution and uptake of liposomal doxorubicin (Caelyx) in human osteosarcoma xenografts. Br.J Cancer.

[38] Diop-Frimpong B, Chauhan VP, Krane S, Boucher Y, Jain RK (2011). Losartan inhibits collagen I synthesis and improves the distribution and efficacy of nanotherapeutics in tumors. Proc.Natl.Acad.Sci.U.S.A.

[39] Reagan-Shaw S, Nihal M, Ahmad N (2008). Dose translation from animal to human studies revisited. FASEB J.

[40] Richer JK, Jacobsen BM, Manning NG, Abel MG, Wolf DM, Horwitz KB (2002). Differential gene regulation by the two progesterone receptor isoforms in human breast cancer cells. J.Biol.Chem.

[41] Valent A, Penault-Llorca F, Cayre A, Kroemer G (2013). Change in HER2 (ERBB2) gene status after taxane-based chemotherapy for breast cancer: polyploidization can lead to diagnostic pitfalls with potential impact for clinical management. Cancer Genet.

[42] Ikeda H, Taira N, Nogami T, Shien K, Okada M, Shien T, Doihara H, Miyoshi S (2011). Combination treatment with fulvestrant and various cytotoxic agents (doxorubicin, paclitaxel, docetaxel, vinorelbine, and 5-fluorouracil) has a synergistic effect in estrogen receptor-positive breast cancer. Cancer Sci.

[43] Pico C, Martin M, Jara C, Barnadas A, Pelegri A, Balil A, Camps C, Frau A, Rodriguez-Lescure A, Lopez-Vega JM, De La HJ, Tres A, Alvarez I, Alba E, Arcusa A, Oltra A, Batista N, Checa T, Perez-Carrion R, Curto J (2004). Epirubicin-cyclophosphamide adjuvant chemotherapy plus tamoxifen administered concurrently versus sequentially: randomized phase III trial in postmenopausal node-positive breast cancer patients. A GEICAM 9401 study. Ann.Oncol.

[44] Skor MN, Wonder EL, Kocherginsky M, Goyal A, Hall BA, Cai Y, Conzen SD (2013). Glucocorticoid receptor antagonism as a novel therapy for triple-negative breast cancer. Clin.Cancer Res.

[45] Institute of Laboratory Animal Resources CoLSNRC (1996). Guide for the Care and Use of Laboratory Animals.

[46] Lanari C, Lamb CA, Fabris VT, Helguero LA, Soldati R, Bottino MC, Giulianelli S, Cerliani JP, Wargon V, Molinolo A (2009). The MPA mouse breast cancer model: evidence for a role of progesterone receptors in breast cancer. Endocr.Relat Cancer.

[47] Jacobsen BM, Richer JK, Schittone SA, Horwitz KB (2002). New Human Breast Cancer Cells to Study Progesterone Receptor Isoform Ratio Effects and Ligand-independent Gene Regulation. J.Biol.Chem.

[48] Sahores A, Luque GM, Wargon V, May M, Molinolo A, Becu-Villalobos D, Lanari C, Lamb CA (2013). Novel, low cost, highly effective, handmade steroid pellets for experimental studies. PLoS. ONE.

[49] Charrois GJ, Allen TM (2004). Drug release rate influences the pharmacokinetics, biodistribution, therapeutic activity, and toxicity of pegylated liposomal doxorubicin formulations in murine breast cancer. Biochim. Biophys. Acta.

[50] Desai N, Trieu V, Yao Z, Louie L, Ci S, Yang A, Tao C, De T, Beals B, Dykes D, Noker P, Yao R, Labao E, Hawkins M, Soon-Shiong P (2006). Increased antitumor activity, intratumor paclitaxel concentrations, and endothelial cell transport of cremophor-free, albumin-bound paclitaxel, ABI-007, compared with cremophor-based paclitaxel. Clin Cancer Res.

[51] Ogston KN, Miller ID, Payne S, Hutcheon AW, Sarkar TK, Smith I, Schofield A, Heys SD (2003). A new histological grading system to assess response of breast cancers to primary chemotherapy: prognostic significance and survival. Breast.

[52] Lizano C, Perez MT, Pinilla M (2001). Mouse erythrocytes as carriers for coencapsulated alcohol and aldehyde dehydrogenase obtained by electroporation in vivo survival rate in circulation, organ distribution and ethanol degradation. Life Sci.

