# Correction: The effectiveness of nano chemotherapeutic particles combined with mifepristone depends on the PR isoform ratio in preclinical models of breast cancer

**DOI:** 10.18632/oncotarget.28842

**Published:** 2026-03-31

**Authors:** Gonzalo Sequeira, Silvia I Vanzulli, Paola Rojas, Caroline Lamb, Lucas Colombo, María May, Alfredo Molinolo, Claudia Lanari

**Affiliations:** ^1^Institute of Experimental Biology and Medicine, IBYME-CONICET, Buenos Aires, Argentina; ^2^National Academy of Medicine, Buenos Aires, Argentina; ^3^Instituto Roffo, Buenos Aires, Argentina; ^4^Oral and Pharyngeal Cancer Branch, NIDCR, NIH, Bethesda, USA; ^*^Both authors had equal participation

**This article has been corrected:** It has been noted that an image in Figure 6B was incorrectly selected, resulting in identical images being shown for both the control tumor and the combo-treated tumor.

While the original digital images underlying this figure are no longer available, the original stained slides remain in the authors’ possession and were presented to the Integrity office. New images have been captured from these original slides, confirming that no morphological differences exist between the control and combo-treated tumors.

The replacement of these images does not alter the study’s conclusions. The authors sincerely apologize for any inconvenience this may cause to the readers.

Original article: Oncotarget. 2014; 5:3246–3260. 3246-3260. https://doi.org/10.18632/oncotarget.1922

**Figure 6:**
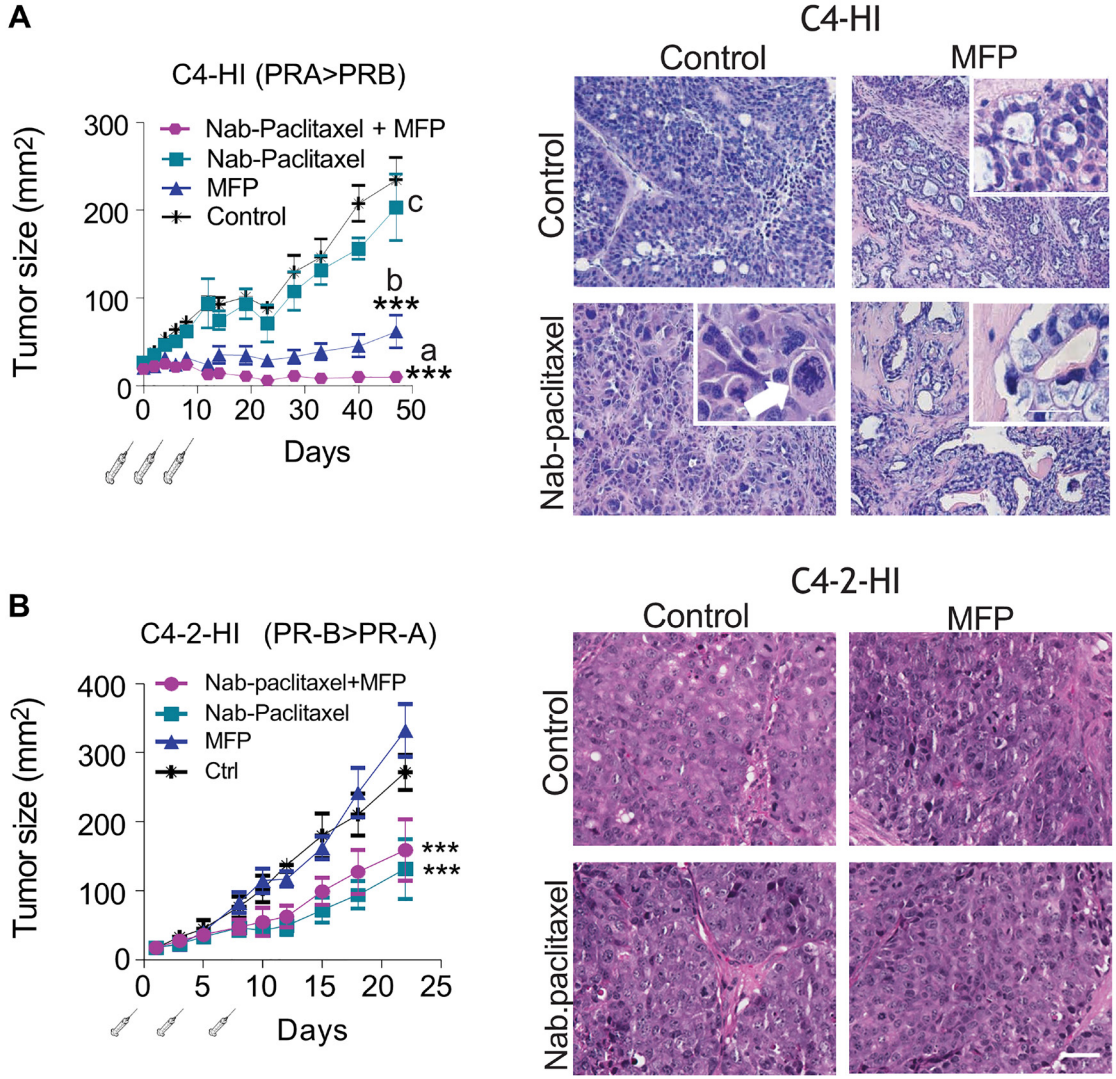
MFP improves the therapeutic effects of Nab-paclitaxel only in mammary carcinomas showing higher levels of PRA than PRB. (**A**) left: nude mice with palpable C4-HI tumors were treated as explained in Materials and Methods with Nabpaclitaxel (30 mg/kg), MFP, or both treatments (*n* = 6/group). MFP induced an inhibitory effect on tumor growth that was greater when combined with Nab-paclitaxel. ^***^p < 0.001 experimental vs. control; a vs. b or c: *p* < 0.001. Right: Representative images of tumors excised 10 days after treatment initiation. Nab-paclitaxel induced an early increase in aberrant cells with multinucleated pleomorphic nuclei showing partially condensed chromatin. The inset shows a tetrapolar mitosis (arrow head) and a mitotic cell with chromosomal spreading (arrow). Eosinophilic cells and cells with large amounts of cytoplasm were also encountered. MFP induced differentiation. In (Nab-paclitaxel+MFP)-treated tumors, the combined treatment mimics the effect of antiprogestin, although a higher number of aberrant signet ring-like cells can be observed (inset). (**B**) left: Nab-paclitaxel (15 mg/kg) inhibited the growth of C4-2-HI, and MFP did not improve the effect of Nab-paclitaxel. Right: No morphological differences were observed in C4-2-HI tumors with the combined treatments. Bar = 40 μm; inset bar = 20 μm.

